# Novel aspects of mast cell and basophil function: Highlights from the 9th meeting of the European Mast Cell and Basophil Research Network (EMBRN)—A Marcus Wallenberg Symposium

**DOI:** 10.1111/all.14065

**Published:** 2019-10-18

**Authors:** Jenny Hallgren, Lars Hellman, Marcus Maurer, Gunnar P. Nilsson, Sara Wernersson, Magnus Åbrink, Gunnar Pejler

**Affiliations:** ^1^ Department of Medical Biochemistry and Microbiology Uppsala University Uppsala Sweden; ^2^ Department of Cell and Molecular Biology Uppsala University Uppsala Sweden; ^3^ Department of Dermatology and Allergy Allergie‐Centrum‐Charité Charité ‐ Universitätsmedizin Berlin Berlin Germany; ^4^ Department of Medicine Solna Karolinska Institutet Stockholm Sweden; ^5^ Department of Medical Sciences Uppsala University Uppsala Sweden; ^6^ Department of Anatomy, Physiology and Biochemistry Swedish University of Agricultural Sciences Uppsala Sweden; ^7^ Department of Biomedical Sciences and Veterinary Public Health Swedish University of Agricultural Sciences Uppsala Sweden

Mast cells and basophils are important members of the immune system. Both of these cell types are implicated in allergic responses, being strongly responsive to activation/degranulation through allergen‐induced crosslinking of IgE molecules bound to their high‐affinity cell surface receptors (FcεRI). However, there have been intense research efforts during the past decades where the roles of mast cells and basophils in many contexts beyond allergy have been explored. These efforts have formed the basis for a much more complex view of the biology of these cell types.^1^ For example, mast cells and basophils are strongly implicated in pruritus, cancer, various autoimmune disorders, chronic inflammation, urticaria, transplantation, wound healing, obesity, infertility and fibrosis.^2^, ^3^, ^4^, ^5^, ^6^, ^7^ In all of these conditions, mast cells and basophils have predominantly detrimental functions, but they are also known to have beneficial functions, most notably in the host protection against bacterial, fungal and parasitic insults, and against envenomation.^7^, ^8^, ^9^

The state‐of‐the‐art regarding these issues was recently discussed during the 9th meeting of the European Mast Cell and Basophil Research Network (EMBRN), held in Uppsala, Sweden (June 17‐19, 2019). The meeting brought together close to 200 researchers in the area of mast cell and basophil biology, with attendees from wide‐spread geographic origins, including Europe, Japan, China, the USA, South America, Mexico, New Zeeland and Australia. Notably, the meeting was attended by foremost scientists at both senior and junior level. The meeting's faculty and participants reflected the high level of diversity of the scientific mast cell and basophil community and were well gender‐balanced, with 48% of the oral presentations given by women. Altogether, 44 oral presentations were given and 57 posters were presented, the latter divided into two well‐attended poster sessions.

**Figure 1:**
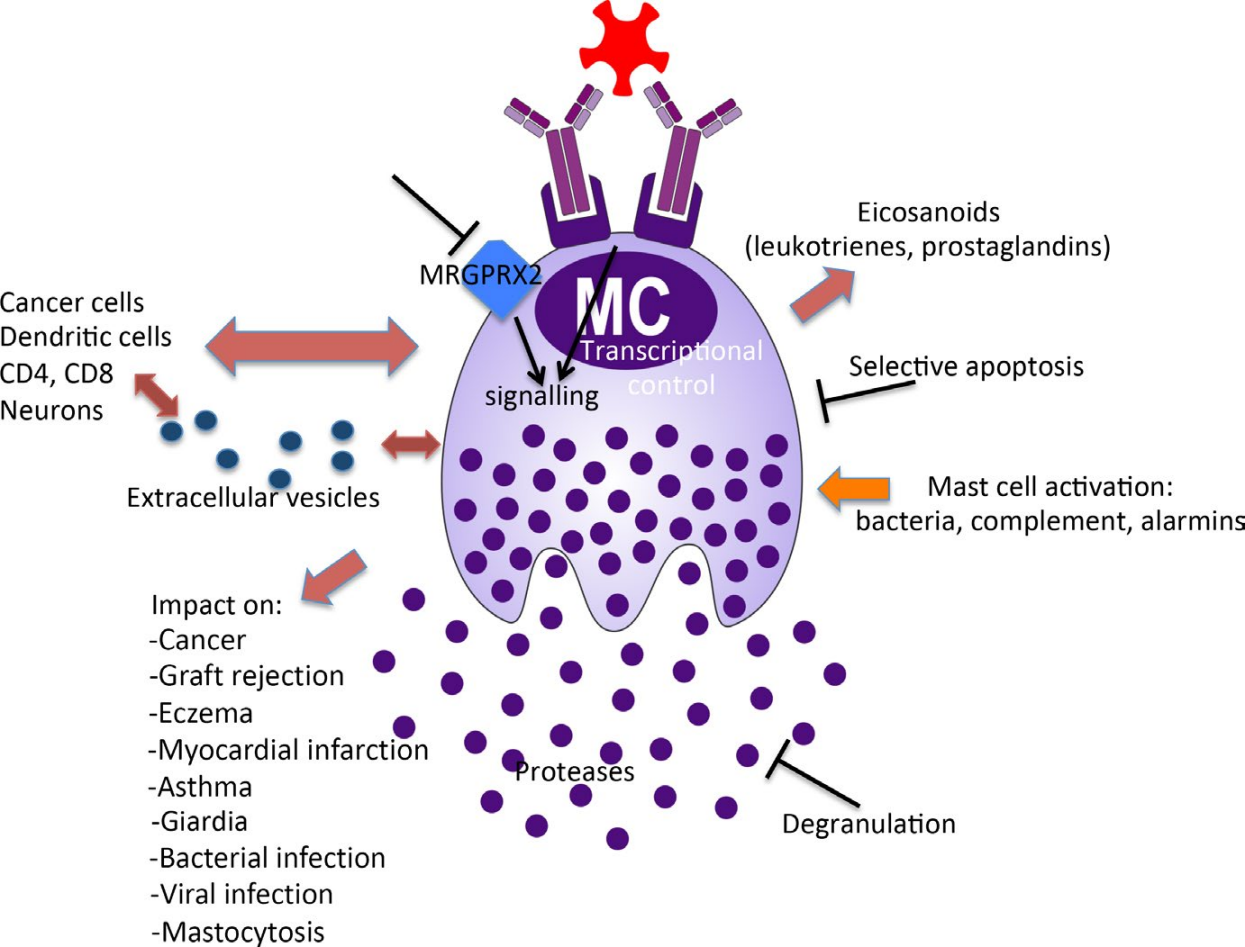
Current trends in mast cell research. The figure depicts major directions in ongoing research

**Figure 2:**
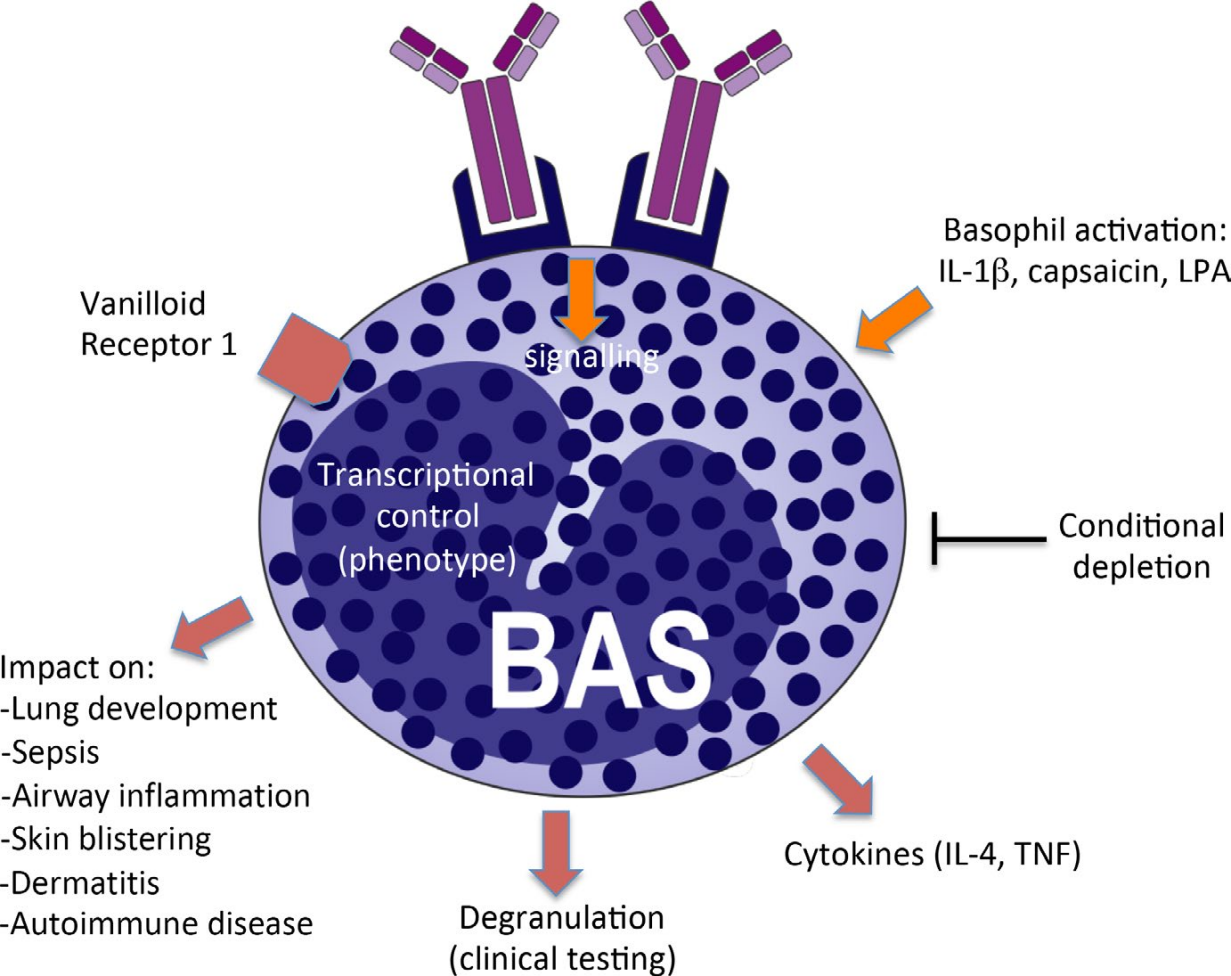
Current trends in basophil research. The figure depicts major directions in ongoing research

The meeting covered multiple novel aspects of mast cell/basophil function, as detailed below and in Figures 1 and 2.

Several presentations were focused on the development and phenotype of mast cells and basophils, and how their phenotypes are affected by either tissue location or by disease conditions such as cancer. Notably, novel studies on this topic have used advanced immunohistochemical technology, 3‐D imaging as well as single‐cell analysis. New insight into the transcriptional control in mast cells was also presented, including epigenetic mechanisms.

Another major issue was the mechanisms of mast cell/basophil activation. Several of the presentations on this topic focused on IgE‐mediated mechanisms, but mast cell activation by novel pathways, such as the MRGPRX2 receptor, alarmins and complement, were also discussed. Signalling mechanisms operating downstream of receptor activation were another area of discussion. A number of presentations were focused on the synthesis of eicosanoids (leukotrienes and prostaglandins) in response to mast cell/basophil activation and on the biological consequences of eicosanoid release.

Several of the presentations revealed newly discovered roles of basophils in various in vivo contexts, including lung development, sepsis, airway inflammation, skin blistering, dermatitis and autoimmune disease. Monitoring of basophil activation for clinical testing was also discussed, as was the cytokine output from activated basophils.

A hot current topic is to decipher how mast cells/basophils communicate with other cell types. This was covered by numerous presentations at the meeting, with a particular focus on the interaction between mast cells and dendritic cells, melanoma cells, neurons, CD4^+^ and CD8^+^ cells. In several of these presentations, the role of extracellular vesicles in cell‐cell communication was discussed.

Several presentations described a role for mast cells in a number of novel settings, including fungal infection, skin rejection, viral infections, myocardial infarction, Giardia infection, Alzheimer's disease, eczema and cancer. New insight into how mast cells and their progenitors affect asthma was also reported. New knowledge of the role of mast cells in regulating bacterial infection and of how mast cells are activated in response to bacterial insult was also presented, along with new findings delineating the interaction between mast cells and the microbiota. Various aspects of mastocytosis and mast cell leukaemia were also discussed. In several of the presentations, the role of the mast cell‐restricted proteases (chymase, tryptase and carboxypeptidase A3) in various settings was addressed.

Another major topic of the meeting was to discuss regimes for interfering with detrimental functions of mast cells/basophils. Quite a few novel strategies for this purpose were presented, including studies of how low molecular weight inhibitors suppress responses downstream of mast cell activation caused by IgE‐dependent and ‐independent mechanisms. Further, anti‐mast cell/basophil therapies using anti‐IgE biologicals such as omalizumab and ligelizumab and treatments that target inhibitory receptors were discussed. Novel strategies for inducing selective mast cell apoptosis were also presented, and mast cell apoptosis as a mechanism of maintaining skin homoeostasis was addressed.

Altogether, the 9th meeting of the (EMBRN) provided a comprehensive update in the field of mast cell/basophil biology. We anticipate that multiple new research initiatives will be taken as a result of the findings presented at the meeting and we also foresee that multiple novel collaborative efforts will be emanating from the meeting, altogether giving rise to joint publications and joint initiatives for grant applications.
